# Serum Levels of miR-143 Predict Survival in Critically Ill Patients

**DOI:** 10.1155/2019/4850472

**Published:** 2019-10-23

**Authors:** Christoph Roderburg, Alexander Koch, Fabian Benz, Mihael Vucur, Martina Spehlmann, Sven H. Loosen, Mark Luedde, Sebastian Rehse, Georg Lurje, Christian Trautwein, Frank Tacke, Tom Luedde

**Affiliations:** ^1^Department of Medicine III, University Hospital RWTH Aachen, Pauwelsstrasse 30, 52074 Aachen, Germany; ^2^Department of Gastroenterology/Hepatology, Charité University Medical Center Berlin, Campus Virchow Klinikum and Charité Mitte, Augustenburger Platz 1, 13353 Berlin, Germany; ^3^Department of Internal Medicine III, University of Kiel, Schittenhelmstrasse 12, 24105 Kiel, Germany; ^4^Department of Internal Medicine, Hospital of Preetz, Am Krankenhaus 5, 24211 Preetz, Germany; ^5^Department of Visceral and Transplantation Surgery, University Hospital RWTH Aachen, Pauwelsstrasse 30, 52074 Aachen, Germany; ^6^Division of Gastroenterology, Hepatology and Hepatobiliary Oncology, University Hospital RWTH Aachen, Pauwelsstrasse 30, 52074 Aachen, Germany

## Abstract

**Background and Aims:**

Recent data suggested a potential role of miR-143 as a biomarker for systemic inflammation and infection. However, its role in critical illness and sepsis is only poorly understood.

**Methods:**

We determined circulating levels of miR-143 in 218 critically ill patients, of which 135 fulfilled sepsis criteria, and compared them to 76 healthy controls. Results were correlated with clinical records.

**Results:**

In the total cohort of critically ill patients from a medical intensive care unit (ICU), miR-143 serum levels tended to be lower compared to healthy control samples, but this difference did not reach statistical significance. In ICU patients, serum levels of miR-143 were independent of disease etiology, including the presence of sepsis, or severity of disease. Importantly, low miR-143 serum levels were associated with an unfavorable short- and long-term prognosis in ICU patients. Our study identified different optimal cut-off values at which low miR-143 serum levels predicted mortality with a high diagnostic accuracy. In line with this, concentrations of circulating miR-143 correlated with markers of organ failure such as creatinine, bilirubin, or lactate in our cohort of critically ill patients.

**Conclusion:**

Low miR-143 serum levels are indicative for an unfavorable short- and long-term prognosis in critically ill patients admitted to a medical ICU. Our data suggest a previously unrecognized role for miR-143 measurements as a novel prognostic marker in critically ill patients.

## 1. Introduction

In the last decades, intensive research activities have been made to identify biomarkers for guiding early therapeutic decisions in critically ill patients during ICU treatment [1]. However, due to a lack in specificity and sensitivity, only very few conventional—protein-based—biomarkers have been integrated into daily clinical routine so far. Since the prognosis of critically ill patients is still unacceptably severe, innovative biomarkers reflecting novel pathophysiological concepts are eagerly awaited to improve the therapy of individual critically ill patients.

MicroRNAs (miRNAs) have been demonstrated to act as regulators of gene expression [2]. miRNAs play a critical role in various physiological and pathophysiological processes including inflammation and bacterial infection [3]. Due to their high stability, miRNAs have been proposed as diagnostic, prognostic, and predictive biomarkers in several human diseases [4]. However, the regulation of miRNAs in the serum of patients with critical illness and sepsis is only poorly understood [5, 6]. As an example, it was recently shown that elevated serum levels of miR-150 might indicate an unfavorable long-term outcome in critically ill patients during ICU treatment [7]. Interestingly, this miRNA was shown to be involved in the regulation of systemic inflammation and bacterial infection [8]. Similar to miR-150, alterations in miR-143 serum levels were recently suggested as a biomarker in the context of critical illness and sepsis [9]. miR-143 is part of the miR-143/miR-145 cluster representing a microRNA cluster involved in the regulation of smooth muscle cell differentiation, leading to a phenotypic switch in response to vascular injury and remodeling [10]. Moreover, alterations in miR-143 expression were found in patients with inflammatory diseases such as inflammatory bowel diseases [11] or cancer [4, 12, 13]. In patients with sepsis, it was shown that, along with other miRNAs, miR-143 was upregulated in the T-cell subpopulation [14]. Based on these observations, we analyzed the diagnostic and prognostic value of miR-143 serum levels in a large cohort of critically ill patients with or without the presence of septic disease who were treated at our medical ICU, as recently described [15].

## 2. Materials and Methods

### 2.1. miRNA Isolation from Serum

400 *μ*l serum was spiked with miScript miRNA mimic SV40 (Qiagen; 2 *μ*M, 1 *μ*l/100 *μ*l serum) for sample normalization. 800 *μ*l phenol (QIAzol) and 200 *μ*l chloroform were added to the sample and mixed vigorously for 15 sec followed by an incubation at room temperature for 10 min. Samples were centrifuged for 15 min at 12,000 g until complete phase separation. The aqueous phase, containing total RNA, was precipitated with 500 *μ*l 100% isopropanol and 2 *μ*l glycogen (Fermentas, St. Leon-Rot, Germany) overnight at -20°C. After centrifugation at 4°C for 30 min (12,000 g), the pellets were washed once with 70% ethanol. Precipitated RNA was resuspended in 30 *μ*l RNase-free water (Ambion, Austin, TX). To assess the quality of RNA, the samples were measured with a NanoDrop spectrophotometer (NanoDrop), and a small RNA assay for Agilent's Bioanalyzer was performed (Agilent Technologies, Böblingen, Germany).

### 2.2. Quantitative Real-Time PCR

Quantitative real-time polymerase chain reaction (PCR) was performed as recently described [2, 15]. In detail, 5 *μ*l of extracted total RNA was used to synthesize complementary deoxyribonucleic acid (cDNA) utilizing a miScript Reverse Transcriptase Kit (Qiagen) according to the manufacturer's protocol and was diluted in suitable amounts of H_2_O. The rest of the protocol was conducted via the miScript Reverse Transcription Kit according to the manufacturer's protocol (Qiagen). cDNA samples (2 *μ*l) were used for quantitative real-time PCR in a total volume of 25 *μ*l using the miScript SYBR Green PCR Kit (Qiagen) and miRNA specific primers (Qiagen, primer sequences available online) on a qPCR machine (Applied Biosystems 7300 Sequence Detection System, Applied Biosystems, Foster City, CA). All results are expressed as 2-*ΔΔ*CT and represent the *x*-fold increase of gene expression compared to the control group. Data were generated and analyzed using the SDS 2.3 and RQ manager 1.2 software packages.

### 2.3. Sampling and Outcome Definitions in Critically Ill Patients

To obtain serum miR-143 levels at the time point of admission to the ICU (before any therapeutic intervention), blood was collected using serum monovettes (Sarstedt, Germany), centrifuged for 8 minutes at 2000 g using a Rotixa 50 centrifuge (Hettich, Germany) following standard protocols within the Labordiagnostisches Zentrum (LDZ) of the university clinic (RWTH) Aachen for patient routine care. No further clearance was performed before RNA isolation. After centrifugation, samples were immediately placed on ice and frozen at -80°C until RNA isolation. Interleukin-6 (IL-6), Interleukin-10 (IL-10), TNF, soluble urokinase plasminogen activator receptor (suPAR), Osteopontin, Glucocorticoid-induced TNF receptor ligand (GITRL), and A proliferation-inducing ligand (APRIL) were measured as described previously (e.g., [16–20]). All other laboratory markers mentioned within this manuscript were measured as part of clinical routine at the Labordiagnostisches Zentrum (LDZ) of the University Hospital (RWTH) Aachen. Glomerular filtration rates (GFR) were calculated on basis of serum cystatin C levels. ICU mortality was defined as death on ICU; overall mortality included death at the ICU or during the observation period (after discharge from the ICU and hospital).

### 2.4. Study Design and Patient Characteristics

In the present study, we enrolled 207 patients that were consecutively admitted to the General Internal Medicine intensive care unit (ICU) at the University Hospital Aachen (Table 1). The clinical course of patients was observed in a follow-up period of three years by directly contacting the patients, the patients' relatives, or their primary care physician. Patients who met the criteria proposed by the American College of Chest Physicians and the Society of Critical Care Medicine Consensus Conference Committee for severe sepsis and septic shock were categorized as sepsis patients, the others as nonsepsis patients [16, 21, 22]. As a control population, we analyzed 76 healthy blood donors (47 males, 29 females, median age 33 years, range 18-67) with normal values for blood counts, C-reactive protein, and liver enzymes.

Patients were included into the study upon providing a written informed consent, and the ethics committees approved this consent procedure. The study protocol was approved by the local ethics committee and conducted in accordance with the ethical standards laid down in the Declaration of Helsinki (ethics committee of the University Hospital Aachen, RWTH University, Aachen, Germany, reference number EK 150/06).

### 2.5. Statistical Analysis

Data are displayed as median and range considering the skewed distribution of most parameters. Differences between two groups were assessed by the Mann-Whitney *U* test, and multiple comparisons between more than two groups have been conducted by the Kruskal-Wallis-ANOVA and Mann-Whitney *U* test for post hoc analysis. Box-plot graphics illustrate comparisons between subgroups and display a statistical summary of the median, quartiles, range, and extreme values. The whiskers extend from the minimum to the maximum value excluding outside and far out values which are displayed as separate points. An outside value (indicated by an open circle) was defined as a value that is smaller than the lower quartile minus 1.5-times the interquartile range or larger than the upper quartile plus 1.5-times the interquartile range. A far out value was defined as a value that is smaller than the lower quartile minus three times the interquartile range or larger than the upper quartile plus three times the interquartile range. All values, including “outliers,” have been included for statistical analyses. Correlations between variables have been analyzed using the Spearman correlation test, and values of *p* < 0.05 were considered statistically significant. The Kaplan-Meier curves were plotted to display the impact on survival. The receiver operating characteristic (ROC) curve analysis and the derived area under the curve (AUC) statistic provide a global and standardized appreciation of the accuracy of a marker or a composite score for predicting an event. ROC curves were generated by plotting sensitivity against 1 − specificity. All statistical analyses were performed with SPSS version 12.0 (SPSS, Chicago, IL, USA).

## 3. Results

### 3.1. miR-143 Serum Levels in Critically Ill Patients and Healthy Controls

**Figure 1:**
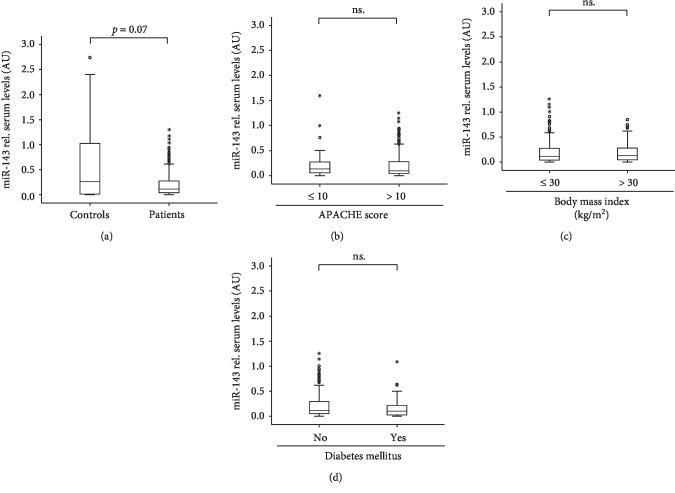
Serum miR-143 levels of critically ill patients at ICU admission. (a) qPCR was used to determine concentrations of circulating miR-143 at admission to the ICU. This analysis revealed a trend toward lower miR-143 concentrations in critically ill patients (*n* = 218) as compared with healthy controls (*n* = 76). (b) Serum miR-143 concentrations were independent of the disease severity. (c) Serum concentrations of miR-143 were similar in patients with or without diabetes mellitus type 2. (d) Serum concentrations of miR-143 were similar in patients with or without obesity.

Based on recent data suggesting a role for miR-143 as a biomarker in the context of critical illness and sepsis [9], we analyzed serum concentrations of miR-143 in 218 critically ill patients and 76 healthy blood donors as a control population. In these analyses, we found a trend towards lower levels of miR-143 in ICU patients compared to control samples (Figure 1(a)). However, due to the large variance in the control group, the difference did not reach statistical significance (*p* = 0.07). Next, we examined whether serum levels of miR-143 reflect disease severity and compared miR-143 serum concentrations between patients with a more severe disease state according to a higher APACHE II score and those with a less severe state of disease (Figure 1(b)). Unexpectedly, no difference in miR-143 serum levels became apparent between both groups. In line with these results, miR-143 levels did not correlate with APACHE II, SAPS2, or SOFA scores in our cohort of critically ill patients (Table 2).

Metabolic comorbidities were shown to influence the outcome of critically ill patients [23]. Since decreased serum levels of miR-143 were recently described in patients with obesity [24], we next analyzed the impact of preexisting type 2 diabetes or obesity in our cohort of critically ill patients, revealing that miR-143 serum concentrations were independent of these comorbidities (Figures 1(c) and 1(d)). In line with this, miR-143 levels did not correlate with routinely used markers of metabolic diseases in our cohort of critically ill patients (Table 2).

### 3.2. miR-143 Serum Levels Are Unaltered in Patients with Sepsis

**Figure 2:**
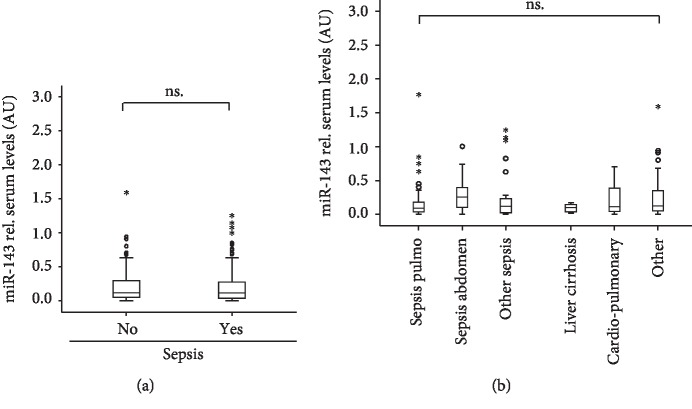
Serum miR-143 concentrations are unaltered in sepsis. (a) miR-143 serum levels were analyzed by qPCR in critically ill patients with sepsis and patients without septic etiology of critical illness. (b) miR-143 serum did not vary between the different etiologies of septic or nonseptic disease.

Our cohort of critically ill patients consisted of 135 patients who fulfilled sepsis criteria and 72 patients with another disease etiology (Table 3). Recently, elevated serum concentrations of miR-143 were found in Asian patients with sepsis (*n* = 103) compared to patients with SIRS (*n* = 95) or healthy controls (*n* = 40). We therefore analyzed miR-143 serum levels in patients with or without septic disease in our cohort of patients. In our ICU cohort, serum levels of miR-143 did not differ between these groups (Figure 2(a)). In line with these results, correlation analyses demonstrated that miR-143 concentrations did not correlate to routinely used sepsis markers such as C-reactive protein (CRP), procalcitonin (PCT), or tumor necrosis factor (TNF) (Table 2). Furthermore, subgroup analyses did not identify an etiology with a specific regulation of miR-143 (Figure 2(b)).

### 3.3. miR-143 Serum Levels Predict ICU Survival in Critically Ill Patients

**Figure 3:**
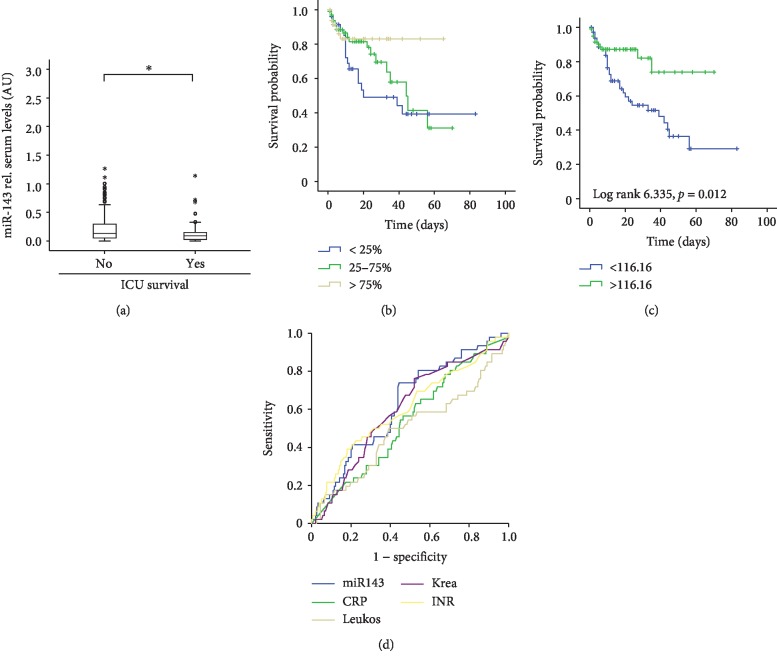
Low serum levels of miR-143 indicate an unfavorable outcome in critically ill patients treated on a medical ICU. (a) Serum levels of miR-143 were analyzed by qPCR in critically ill patients that survived their ICU stay or succumbed to death. Patients that survived had significantly higher miR-143 serum levels on admittance to the ICU than those patients that succumbed to death. (b) Kaplan-Meier survival curves of ICU patients are displayed, showing that patients with miR-143 levels within the lowest quartile of all patients displayed the lowest short-term survival at the ICU. (c) The Youden index was used to calculate the optimal threshold to distinguish between survivors and patients that died during ICU treatment. The Kaplan-Meier survival curve analyses revealed that patients with miR-143 below this threshold displayed a significantly impaired prognosis. Significances are given in the figure. (d) The ROC curve analysis comparing the prognostic value of miR-143 with that of other markers routinely accessed in the context of critical illness.

To test whether circulating miR-143 might be useful to predict treatment survival in critically ill patients, we next analyzed serum levels of miR-143 in patients that succumbed to death during ICU treatment and those who survived. Interestingly, patients who survived their ICU stay displayed significantly higher levels compared to those who died (Figure 3(a)). Similarly, Kaplan-Meier curve analyses revealed that patients with low miR-143 levels (below 116.16 AU) showed a significantly impaired survival probability at the ICU (Figure 3(b)). To substantiate these findings, we next applied the approach of Ray et al. [25] to determine an optimal threshold with the highest Youden index for miR-143 levels predicting the patients' survival during ICU treatment. This analysis revealed a relative miR-143 concentration of 116.16 (AU) for the best sensitivity and specificity to decide whether a patient will survive or not. Using this optimal cut-off value, we performed the Kaplan-Meier curve analysis, showing that patients with high miR-143 serum concentrations above the cut-off value had a more favorable prognosis compared to those with lower values (Figure 3(c)). Finally, we hypothesized that low levels of miR-143 could discriminate between critically ill patients that survive ICU treatment and those that do not. Therefore, we attempted to compare its predictive accuracy with other laboratory parameters routinely accessed in the context of critical illness. The ROC curve analysis revealed higher AUC statistics for miR-143 (AUC = 0.628) compared to CRP (AUC = 0.563), the leukocyte count (AUC = 0.491), creatinine (AUC = 0.599), or the INR value (0.604) (Figure 3(d)).

### 3.4. miR-143 Serum Levels Predict Overall Survival in Critically Ill Patients

**Figure 4:**
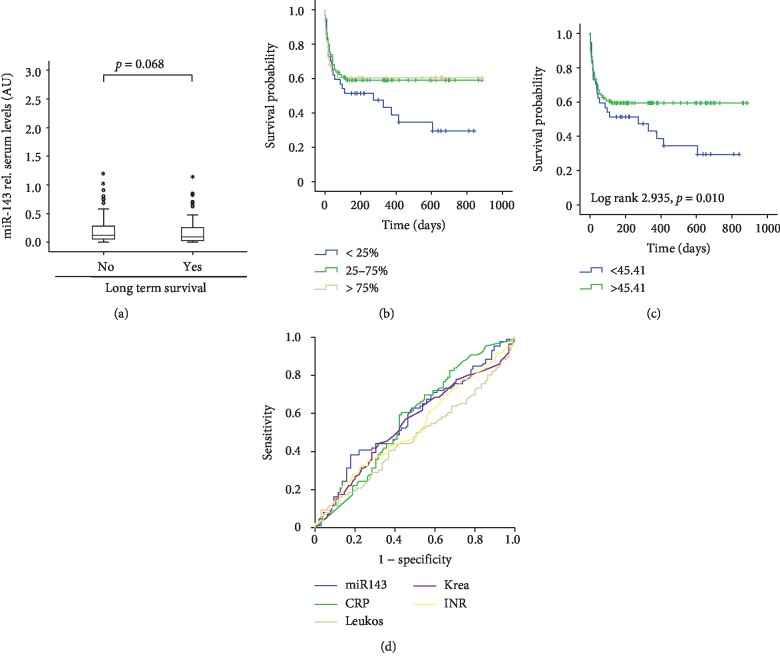
Low miR-143 serum concentrations are associated with an impaired long-term prognosis of critically ill patients. (a) Patients that succumbed to death during long-term follow-up had lower serum miR-143 levels on admittance to the ICU (*p* = 0.063, *U*-test) compared to patients that survived. (b) Kaplan-Meier curve analysis revealing that patients with miR-143 concentrations within the lower quartile had an increased overall mortality as compared to patients with miR-143 serum concentrations of the highest quartile. (c) The Youden index was used to calculate the optimal threshold to distinguish between survivors and patients that died during long-term follow-up. The Kaplan-Meier survival curve analyses revealed that patients with miR-143 below this threshold displayed a significantly impaired prognosis. (d) ROC curve analysis comparing the prognostic value of miR-143 with that of other markers routinely accessed in the context of critical illness.

Since many of the patients died after initially being successfully discharged from the ICU, we subsequently analyzed miR-143 serum levels in patients that died during long-term follow-up and patients who survived. Of note, this analysis revealed that survivors demonstrated significantly higher miR-143 levels than patients who died during long-term follow-up (Figure 4(a)). Consequently, the Kaplan-Meier curve analysis revealed that patients with lower levels of circulating miR-143 displayed an impaired long-term prognosis compared to patients with higher miR-143 levels (Figure 4(b)). We again applied the Youden index to determine the optimal threshold of circulating miR-143 to predict overall survival in our cohort of critically ill patients, revealing that a miR-143 value of 45.41 (AU) allows best to distinguish between patients that survived and those that died during the long-term follow-up (Figure 4(c)). Similar to our previous analyses, low miR-143 concentrations were associated with an impaired prognosis. We finally performed the ROC curve analysis to compare the predictive value of circulating miR-143 regarding the patients' overall survival with other routine laboratory parameters or prognostic scores routinely accessed in the context of critical illness. The ROC curve analysis revealed higher AUC statistics for miR-143 (AUC = 0.574) compared to CRP (AUC = 0.568), the leukocyte count (AUC = 0.472), creatinine (AUC = 0.548), or the INR value (0.525) (Figure 4(d)).

### 3.5. miR-143 Serum Levels Are Associated with Markers of Organ Dysfunction in Critically Ill Patients

To identify mechanisms involved in the regulation of miR-143 in critically ill patients, we performed correlation analyses between miR-143 and a broad panel of laboratory markers assessed in clinical routine. While concentrations of miR-143 did not correlate with markers of inflammation or bacterial infection (Table 2), we found a strong correlation between miR-143 and indicators of organ failure in critical illness. In detail, serum levels of miR-143 correlated with a decreased renal function assessed by the glomerular filtration rate (GFR) of cystatin C (*r* = 0.289, *p* = 0.001), elevated creatinine (*r* = −0.254, *p* < 0.001), and urea serum concentrations (*r* = −0.294, *p* < 0.001). In addition to renal dysfunction, miR-143 concentrations significantly correlated with markers of liver injury such as aspartate aminotransferase (AST; *r* = 0.159, *p* = 0.026), alanine aminotransferase (ALT; *r* = 0.206, *p* = 0.003), glutamate dehydrogenase (GLDH; *r* = 0.150, *p* = 0.039), and bilirubin (*r* = 0.185, *p* = 0.035) (Supplementary [Supplementary-material supplementary-material-1]). Moreover, miR-143 concentrations correlated with elevated lipase and amylase serum concentrations as indicators for the presence of acute pancreatitis. In line with this association between miR-143 and organ failure, circulating miR-143 also correlated with serum levels of lactate (*r* = −0.173, *p* = 0.014), cardiac dysfunction (BNP; *r* = −0.383, *p* < 0.001), and the patients' survival time (*r* = −0.348, *p* = 0.006).

## 4. Discussion

Despite continuous advances in diagnostic modalities, triage, and therapeutic management, critically ill patients still represent a major clinical challenge. In this context, besides specific triage systems, various laboratory markers potentially allowing decisions about patients' treatment and clinical course were proposed. As such, next to routinely used markers (e.g., CRP or PCT; [26]), a variety of different experimental protein-basedmarkers such as A proliferation-inducing ligand (APRIL), suPAR, and Osteopontin [17, 27–29] were tested. However, the lack of prognostic sensitivity or specificity for such protein-based markers as well as marker-specific confounding parameters (like sepsis) hampers the translation into routine clinical algorithms that could be applied to heterogeneous patient populations [26, 30]. Compared to “conventional” protein-based markers, circulating miRNAs harbour several advantages: circulating miRNAs are extraordinarily stable towards conditions that usually would degrade most proteins in serum or blood [31]. Moreover, miRNAs are much less complex than most other biological biomarkers [32]. Therefore, many authors hypothesized that circulating miRNAs might perform better in the detection of sepsis or prognosis prediction in critically ill patients. As an example, miR-150 levels were found to be downregulated in patients with urosepsis [33] and predictive for an impaired patients' survival in a cohort of critically ill patients comprising various disease etiologies and severities [15]. In the present study, we demonstrate that miR-143 serum levels appear rather reduced in patients with critical illness when compared to healthy controls and unchanged between patients with septic disease etiology and patients with SIRS. Our results are partially in contrast to previously published results demonstrating elevated levels of miR-143 in patients with sepsis [9]. On the one hand, this might be related to differences in the patient cohorts (unselected critically ill medical patients in our work) or the time point of sampling; on the other hand, technical aspects (method of sample collection, data normalization, and analysis) might also account for diverging findings. In this respect, it is important to note that in the context of critical illness and sepsis, the interstudy variances in terms of miRNA regulation patterns are enormous and the fact that different studies show even opposing results with respect to the deregulation of miRNA levels is not uncommon [7, 34, 35]. In an attempt to avoid these biases, we had implemented strict protocols for sample collection and handling in the present study sample. Moreover, in our study, analyses were normalized using spiked-in RNA, which is regarded as the “gold standard” by most authors [36–38]. Finally, the cut-offs for Kaplan-Meier curve analysis were defined using the broadly accepted Youden index, potentially providing different cut-offs compared to previously published studies [15, 35, 39, 40]. These principles might help to overcome technical challenges of miRNA analysis from serum or plasma, giving rise to the expectation that circulating miRNAs might become novel, highly attractive biomarkers in the context of critical illness. Nevertheless, many technical aspects in the context of RNA isolation from serum remain unsolved. As an example, hemolysis may occur during sample handling and bias results. In our analysis, miR-143 concentrations did neither correlate with sodium nor with LDH serum concentrations and only very weakly with bilirubin serum levels, representing markers for hemolysis.

Alterations in miR-143 expression have recently been described in the context of carcinogenesis and cancer progression [41]. Several studies have analyzed the role of circulating miR-143 as a biomarker in malignant diseases. Just recently, it was demonstrated that patients with acute myeloid leukemia and esophageal adenocarcinoma, disease states which are associated with the activation of immune cells, display decreased serum levels of miR-143 [42, 43], which is in line with our results. Moreover, miR-143 might be directly involved in systemic inflammation and defending bacterial infection. As examples, it was demonstrated that miR-143 inhibits Propionibacterium acnes-mediated inflammatory response in the skin and that miR-143 is downregulated in chronic ulcerative colitis, where it might contribute to inflammation colitis-associated carcinogenesis [11]. Most importantly, miR-143 was upregulated in leukocytes after LPS injection in humans [44]. Interestingly, these observations were not in line with a similar regulation of circulating miR-143 in our large collective of ICU patients, since miR-143 showed only a nonsignificant trend towards lower levels in critically ill patients compared to controls. However, we detected lower levels of miR-143 in patients who succumbed to death during ICU treatment, when compared to patients that survived. More importantly, in our analysis, lower levels of miR-143 were significantly associated with an unfavorable short- and long-term prognosis and miR-143 predicted patients' outcome with a higher accuracy than classical markers of organ failure such as creatinine or INR. It was recently suggested that miR-143 is involved in metabolic processes such as insulin tolerance and type II diabetes mellitus. Moreover, it was shown that aerobic exercise can prevent type II diabetes mellitus by downregulating miR-143. However, at least in our cohort of critically ill patients, no direct correlation between diabetes and miR-143 serum levels was found. Notably, since we have not systematically assessed complications of diabetes in our database, we cannot exclude that there might be an association between miR-143 levels and diabetes complications.

Our study bears several limitations and potential bias such as interpretation/selection bias [45] and the lack of pathophysiological mechanisms explaining the regulation of miR-143 in critical illness/sepsis. However, we clearly demonstrate that circulating miR-143 might be indicative for patient prognosis. miR-143 levels upon admission were closely associated with ICU and long-term mortality. Reduced miR-143 concentration indicated an unfavorable prognosis. Furthermore, it is important to note that changes in miR-143 serum concentrations have been described in numerous pathological conditions (see above). Therefore, the use of a specific marker for the diagnosis of sepsis seems to be unattractive at present. Nevertheless, our data suggest that measurements of miR-143 might represent a novel tool to estimate prognosis of critically ill patients and should give rise to further research in order to validate our results in larger and prospective studies on critically ill patients.

## 5. Conclusions

- miR-143 serum concentrations were not altered in samples from critically ill patients taken at admission to the ICU when compared to those from healthy blood donors as controls
- Low miR-143 serum concentrations were lower in critically ill patients that succumbed to death compared to survivors and predicted an unfavorable outcome with higher accuracy

## Figures and Tables

**Table 1:** Baseline patient characteristics.

Parameter	All patients
Number	207
Sex (male/female)	135/72
Age median (range) (years)	63 (18-89)
APACHE II score median (range)	17 (2-43)
SAPS2 score median (range)	43.0 (0-79)
ICU days median (range)	7 (1-83)
Death during ICU (%)	22.2%
Death during ICU or follow-up (%)	42.5%
Body mass index (BMI)	26.78 (16.6-86.5)
Creatinine	1.3 (0-15)
Albumin	27.3 (15.2-52.2)
WBC median (range) (×10^3^/*μ*l)	12.15 (0.1-67.4)
CRP median (range) (mg/dl)	95.5 (<5-230)
Procalcitonin median (range) (*μ*g/l)	0.7 (0-180.6)
Interleukin-6 median (range) (pg/ml)	105 (0-83,000)
Tumor necrosis factor median (pg/ml)	19 (4.9-140)

APACHE: Acute Physiology and Chronic Health Evaluation; CRP: C-reactive protein; ICU: intensive care unit; SAPS: simplified acute physiology score; WBC: white blood cell count.

**Table 2:** Correlations of miR-143 serum concentrations with other laboratory markers. NA: not.

	miR-143 at admission vs. laboratory markers at admission day	
	*r*	*p*
*Markers of inflammation*		
CRP	0.028	0.688
Procalcitonin	0.024	0.771
TNF	-0.029	0.840
IL-10	0.153	0.101
IL-6	0.146	0.142
suPAR	-0.102	0.229
Osteopontin	-0.037	0.783
APRIL	0.038	0.588
GITRL	0.080	0.255
*Markers of organ function*		
Creatinine	-0.254	<0.001
GFR cystatin C	0.289	0.001
Urea	-0.294	<0.001
AST	0.159	0.026
ALT	0.206	0.003
GLDH	0.150	0.039
Bilirubin total	0.185	0.035
GGT	-0.051	0.466
Albumin	0.043	0.965
Lactate	-0.173	0.014
BNP	-0.383	<0.001
*Clinical scoring*		
Apache II	-0.035	0.642
SOFA	-0.042	0.612
*Other*		
Fibrinogen	-0.127	0.303
INR	0.105	0.137
Survival time	-0.348	0.006

**Table 3:** Disease etiology of the study population.

	Sepsis	Nonsepsis
	*n* = 128	*n* = 79
*Sepsis critical illness,n(%)*		
Source of infection		
Pulmonary	68 (32.8%)	
Abdominal	28 (13.5%)	
Urogenital	3 (1.4%)	
Other	29 (14.0%)	
*Nonsepsis critical illness,n(%)*		
Cardiopulmonary disease		28 (13.5%)
Decompensated liver cirrhosis		11 (5.3%)
Nonsepsis other		40 (19.3%)

## Data Availability

The data used to support the findings of this study are restricted by the ethics committee of the university clinic (RWTH) Aachen to protect patient privacy. Data are available upon meaningful request from med3@ukaachen.de for researchers who meet the criteria for access to confidential data.

## Supplementary Materials

Supplementary Materials Correlation analysis of miR-143 serum levels and different laboratory parameters.
