# Supplementary figures and images for: Serum Levels of miR-143 Predict Survival in Critically Ill Patients
Source: Dis Markers. 2019 Oct 23;2019:4850472. doi: 10.1155/2019/4850472 (PMC6854254; doi:10.1155/2019/4850472)

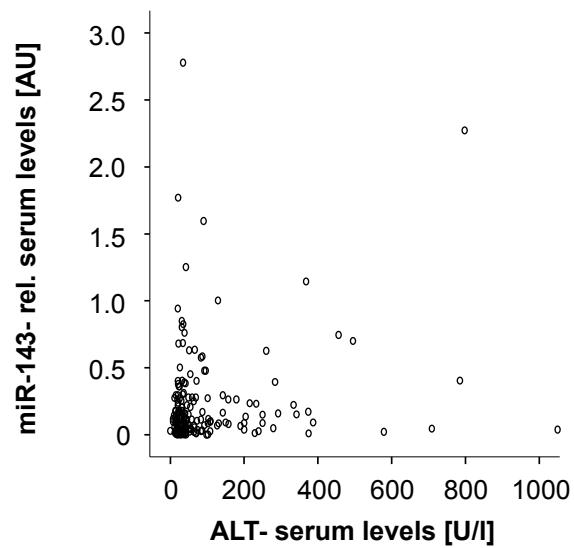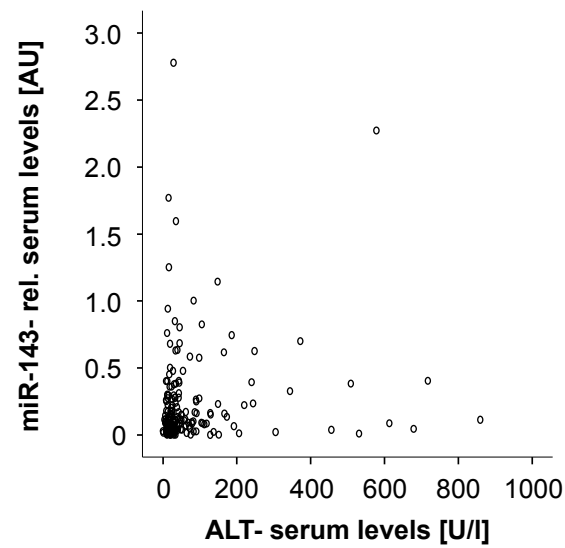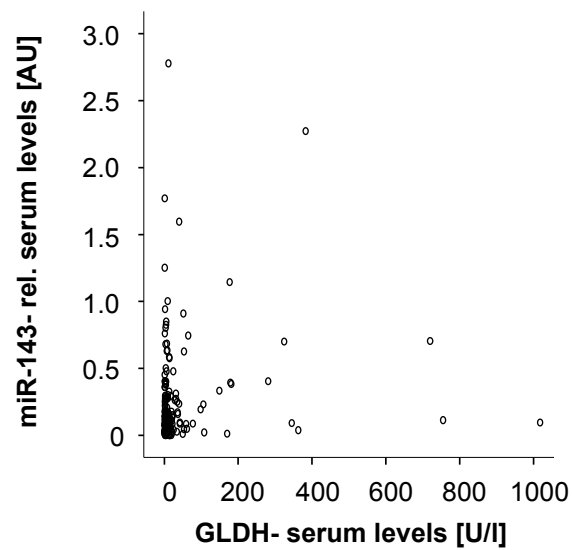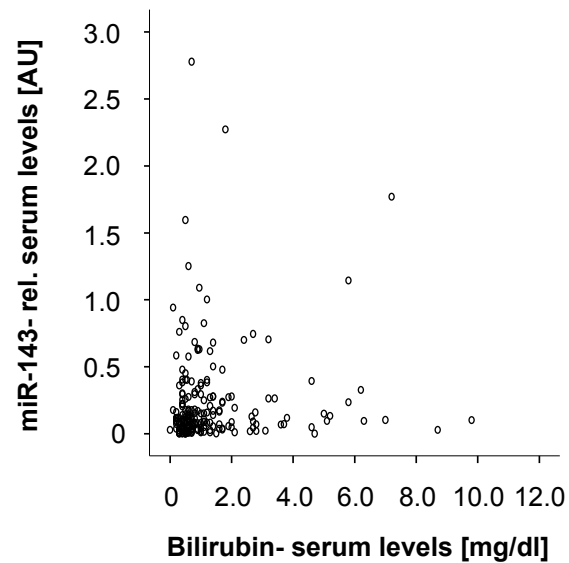

Supplement: Supplementary Materials — Correlation analysis of miR-143 serum levels and different laboratory parameters. [file 4850472.f1.pdf]
